# Sevoflurane reduces clinical disease in a mouse model of multiple sclerosis

**DOI:** 10.1186/1742-2094-9-272

**Published:** 2012-12-19

**Authors:** Paul E Polak, Randall O Dull, Sergey Kalinin, Anthony J Sharp, Richard Ripper, Guy Weinberg, David E Schwartz, Israel Rubinstein, Douglas L Feinstein

**Affiliations:** 1 Department of Anesthesiology, University of Illinois, Chicago, IL, 60612, USA; 2 Jesse Brown Veterans Affairs Medical Center, Chicago, IL, 60612, USA; 3 Department of Medicine, University of Illinois, Chicago, IL, 60612, USA

**Keywords:** Myelin, Experimental autoimmune encephalomyelitis, Inhaled anesthetic, Multiple sclerosis

## Abstract

**Background:**

Inhalational anesthetics have been shown to influence T cell functions both *in vitro* and *in vivo*, in many cases inducing T cell death, suggesting that exposure to these drugs could modify the course of an autoimmune disease. We tested the hypothesis that in mice immunized to develop experimental autoimmune encephalomyelitis (EAE), a well established model of multiple sclerosis (MS), treatment with the commonly used inhalational anesthetic sevoflurane would attenuate disease symptoms.

**Methods:**

C57Bl6 female mice were immunized with myelin oligodendrocyte glycoprotein (MOG) peptide residues 35 to 55 to induce a chronic demyelinating disease. At day 10 after immunization, the mice were subjected to 2 h of 2.5% sevoflurane in 100% oxygen, or 100% oxygen, alone. Following treatment, clinical scores were monitored up to 4 weeks, after which brain histology was performed to measure the effects on astrocyte activation and lymphocyte infiltration. Effects of sevoflurane on T cell activation were studied using splenic T cells isolated from MOG peptide-immunized mice, restimulated *ex vivo* with MOG peptide or with antibodies to CD3 and CD28, and in the presence of different concentrations of sevoflurane. T cell responses were assessed 1 day later by 3-(4,5-dimethylthiazol-2-yl)-2,5-diphenyltetrazolium bromide (MTT) assay for proliferation, lactate dehydrogenase (LDH) release for cell death, and inflammatory activation by production of interleukin (IL)-17 and interferon (IFN)γ.

**Results:**

Clinical scores in the oxygen-treated group increased until day 28 at which time they showed moderate to severe disease (average clinical score of 2.9). In contrast, disease progression in the sevoflurane-treated group increased to 2.1 at day 25, after which it remained unchanged until the end of the study. Immunohistochemical analysis revealed reduced numbers of infiltrating leukocytes and CD4^+^ cells in the CNS of the sevoflurane-treated mice, as well as reduced glial cell activation. In splenic T cells, low doses of sevoflurane reduced IFNγ production, cell proliferation, and increased LDH release.

**Conclusions:**

These results are the first to show attenuation of EAE disease by an inhaled anesthetic and are consistent with previous reports that inhaled anesthetics, including sevoflurane, can suppress T cell activation that, in the context of autoimmune diseases such as MS, could lead to reduced clinical progression.

## Background

Accumulating data suggests that inhaled anesthetics (IAs; this includes sevoflurane, isoflurane, and desflurane) can exacerbate inflammation and lead to neuronal damage in older patients and patients suffering from Alzheimer’s disease (AD). As an example, sevoflurane increased caspase activation and apoptosis, altered amyloid precursor protein (APP) processing, and increased β-amyloid levels in the brains of transgenic mouse models of AD [1] and in APP-expressing cells [1,2]. These studies were based on observations that small molecular weight hydrophobic alkanes can shift oligomerization of proteins such as β-amyloid towards higher oligomers and increase cytotoxicity [3-5]. However, it has been known for many years that certain IAs modulate inflammatory responses in peripheral cells and tissues; although usually associated with suppression of cell activation, in some cases IAs increase inflammatory responses [6].

Multiple sclerosis (MS) is an autoimmune disease that affects approximately 400,000 people in the US and over 2 million people worldwide. The exact causes of MS are not yet precisely known, however, it is well established that activation of the adaptive immune system leads to activation and proliferation of T cells that can infiltrate the central nervous system and release cytokines that lead to oligodendrocyte damage, demyelination, and axonal damage. The infiltrating cells also induce inflammatory activation of resident glial cells, including astrocytes and microglia, which can propagate and maintain disease pathology. Several treatments which reduce T cell activation, proliferation, infiltration into the CNS, or cytokine production are currently used to reduce MS symptoms; however, additional non-invasive, safe methods to modulate T cell functions still have potential value for MS treatment.

In this regard, IAs have been shown to exert significant effects on T cells [7-13], influencing their adhesion properties [14,15] and modifying their inflammatory responses [12,16-19]. It is thought that regional anesthesia can improve postoperative recovery by reducing immunosuppression [20]*.* For example, in normal adult male mice, 40 minutes with sevoflurane increased the total number of CD4+ lymphocytes in the spleen [12]; and *in vitro* sevoflurane increased P-selectin expression and platelet:leukocyte adherence in whole blood [14]; and induced activation of several signaling factors (apoptosis signal-regulating kinase 1 (ASK1), mitogen-activated protein kinase kinase (MAPKK)3 and 6; activating transcription factor 2 (ATF2), and p38 MAPK) in human Jurkat cells [21]. There are also reports that IAs reduce T cell activation or activity; for example both sevoflurane and isoflurane induced apoptosis in whole peripheral blood mononuclear cells (PBMCs) [11]; and desflurane reduced cell adhesion molecule expression in human endothelial cells [22].

These above findings suggest that administration of IAs could impact the course of an autoimmune disease such as MS. However, the possible effects of IAs on the progression of MS symptoms or pathology have not been characterized. A few case reports suggest that sevoflurane does not worsen immediate postoperative recovery [23-25]; however, there are no publications testing either acute or delayed affects of IAs in animal models of MS. In view of the above findings we hypothesized that IA exposure would influence the clinical course of disease in experimental autoimmune encephalomyelitis (EAE), a well characterized model of MS. Our findings indicate that sevoflurane attenuates the progression of clinical disease in EAE mice, which may be due to suppression of T cell activation.

## Methods

### Materials

General chemicals and reagents were from Sigma (St Louis, MO, USA). Secondary antibodies were from Vector Labs (Burlingame, CA, USA). Myelin oligodendrocyte glycoprotein peptide residues 35 to 55 (MOG_35-55_; MEVGWYRSPFSRVVHLYRNGK) was purchased from Anaspec (San Jose, CA, USA).

#### Mice

Female C57BL/6 mice aged 6 to 8 weeks were purchased from Charles River Breeding (Cambridge, MA, USA). Mice were housed five per cage, and kept in a controlled 12 h light/12 h dark environment and provided food *ad libitum*. All animal procedures were approved by the local Institutional Animal Care and Use Committee (IACUC) committee.

#### Induction of EAE

EAE was actively induced in using synthetic MOG_35-55_ as described previously [26]. Mice were injected subcutaneously (two 100 μl injections into adjacent areas in one hind limb) with an emulsion of 300 μg MOG_35-55_ dissolved in 100 μl phosphate-buffered saline (PBS), mixed with 100 μl complete Freund’s adjuvant containing 500 μg of *Mycobacterium tuberculosis* (Difco, Detroit, MI, USA). Immediately after MOG_35-55_ injection, the animals received an intraperitoneal injection of pertussis toxin (PT; 200 ng in 200 μl PBS). Then, 2 days later the mice received a second PT injection, and 1 week later they received a booster injection of MOG_35-55_. This protocol leads to an incidence of >90%, low mortality, average clinical signs between three and four (one or two hindlimbs with paresis or paralysis), lasting disease with no recovery for up to 3 months; frank demyelination in the spinal cords and cerebellum; and neuronal damage by 2 months. Clinical signs were scored on a five-point scale: grade 0, no clinical signs; 1, limp tail; 2, impaired righting; 3, paresis of one hind limb; 4; paresis of two hind limbs; 5, death. When a mouse died it was assigned a score of 5, and that score was carried through for the rest of the study for statistical analysis. Scoring was performed at the same time each day by an investigator blinded to allocation.

#### Treatment with sevoflurane

At 10 days after the booster immunization, at which point mice begin to show clinical signs, mice were subjected to 2 h 2.5% sevoflurane in 100% oxygen, or as control to 2 h of 100% oxygen. Anesthetics and oxygen were provided to mice as a group in a glass chamber. The gas pressure was continuously monitored. After 2 h, the mice were allowed to recover and returned to home cages and monitored for a further 4 weeks. At the end of the study the mice were killed to prepare brain sections for histology and immunocytochemical staining.

#### Tissue preparation and immunohistochemistry

Mouse brains were fixed in 4% paraformaldehyde in 0.1 M phosphate buffer pH 7.6 overnight at 4°C. Dehydration, embedding, paraffin removal, and sectioning were performed using standard protocols as described [27]. Serial sagittal sections (8 μm) were obtained by starting from the midline and included the cerebellum. Following paraffin removal, antigen retrieval was accomplished by boiling in 10 mM citrate buffer for 10 minutes, then blocking with 5% normal donkey serum. Sections were incubated at 4°C overnight with primary antibodies diluted in 1% normal donkey serum: rat monoclonal anti-human glial fibrillary acidic protein (GFAP) B2.210 at 1:300 [28]. After washing, sections were incubated 1 h at 37°C with donkey anti-rabbit rhodamine red-X (RRX) conjugated or donkey anti-rat conjugated with fluorescein isothiocyanate (FITC) secondary antibodies. Sections were washed, fixed with 3.7% formaldehyde in phosphate buffered saline (PBS), quenched in 50 mM ammonium chloride in PBS for 15 minutes, then final washes performed in PBS with 400 ng/ml 4′,6-diamidino-2-phenylindole (DAPI) included in the second wash. Vectashield mounting fluid (Vector Laboratories Inc.) was placed onto sections prior to cover slips placed.

#### Infiltrating cells

Histological examination for infiltrating cells was performed by staining deparaffinized, washed sections with hematoxylin and eosin (H&E). Serial sections through the cerebellum of each mouse were examined for infiltrates and the number of large (>5,000 μm^2^) or small (<5,000 μm^2^) areas of infiltration counted.

#### Image analysis

Images were obtained on a Zeiss Axioplan 2 microscope using an MRm Axiocam for image acquisition and densitometric analysis conducted using Axiovision version 4.5 software (Carl Zeiss Inc. Thornwood, NY, USA). Image acquisition was conducted on sections stained simultaneously and exposed for identical amounts of time. Quantitation of GFAP staining was performed using an object area cutoff of 10 μm^2^ to include cell bodies and processes. The data were analyzed to determine the total area covered by positively stained objects presented as a percentage of the total field of view.

#### Splenic T cell isolation and analyses

Splenocytes were isolated from mice 10 days after the booster MOG immunization. After lysis of red blood cells, splenocytes were plated into 24 well plates at a density of 2 × 10^5^ cells per well in 400 μl RPMI media containing 10% fetal calf serum. The cells were restimulated with MOG_35-55_ peptide (20 μg/ml), or the T cell receptor (TCR) directly activated with rat monoclonal anti-CD3 (0.20 μg/ml) and anti-CD28 (0.5 μg/ml) antibodies. Cells were incubated with indicated concentrations of sevoflurane or equivalent amount of vehicle. After 1 day, aliquots of the media were assayed for levels of interleukin (IL)-17 and interferon (IFN)γ by ELISA following the manufacturer’s instructions (eBioscience, San Diego, CA, USA). Cell proliferation was assessed indirectly using the 3-(4,5-dimethylthiazol-2-yl)-2,5-diphenyltetrazolium bromide (MTT) assay to measure mitochondrial content; and cell viability after 24 h by measurement of lactate dehydrogenase (LDH) released into the media (Promega, Madison, WI, USA).

#### Data analysis

Comparison of clinical signs over time in one group was performed via one-way, non-parametric analysis of variance (ANOVA) (Kruskal-Wallis test) followed by Dunn’s multiple comparison tests. Comparison of the effect of treatment versus control on the development of clinical signs was performed via two-way repeated measures ANOVA. Two group comparisons were performed by Mann–Whitney non-parametric unpaired t tests. Effects of sevoflurane on T cell parameters were compared by parametric one-way ANOVA followed by Tukey *post hoc* comparisons. In all cases significance was taken at *P* <0.05.

## Results

### Sevoflurane attenuates development of clinical signs of EAE

**Figure 1:**
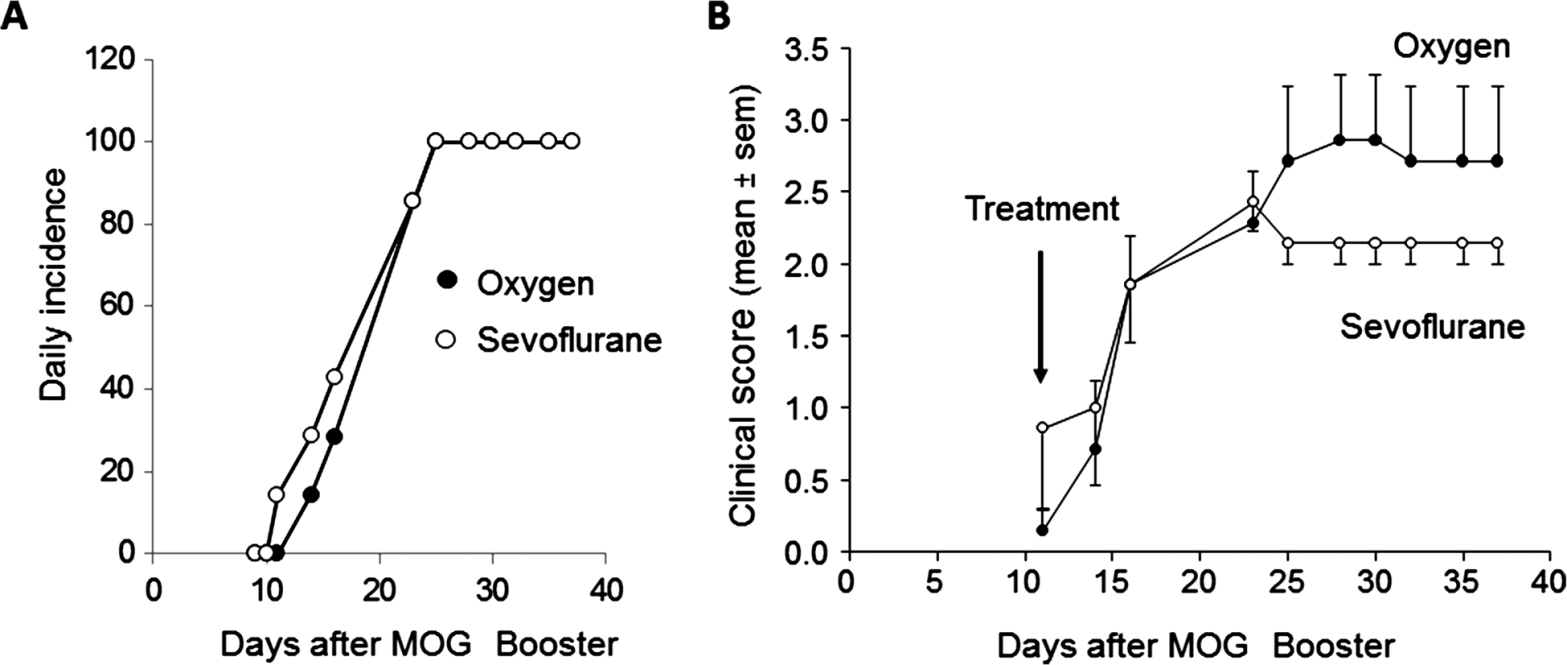
**Sevoflurane attenuates development of clinical scores in experimental autoimmune encephalomyelitis (EAE).** Female C57Bl/6 mice were immunized with myelin oligodendrocyte glycoprotein (MOG)_35–55_ peptide to develop EAE as described in the Methods section. At 10 days after the MOG booster, the mice were subjected to 2 h of 2.5% sevoflurane in 100% oxygen (open circles), or 100% oxygen alone (filled circles). Clinical scores were monitored for the next 4 weeks. (**A**) Daily incidence of disease over the course of the study for both groups. (**B**) Mean ± SEM of average clinical scores of one experiment performed with n = 7 mice per group; similar results were obtained in a second independent study. F[9,1] = 1.98, *P* = 0.049, two-way repeated measures analysis of variance (ANOVA).

C57Bl6 mice were immunized with MOG_35-55_ peptide to develop a chronic demyelinating disease using a standardized protocol. At day 10 after the booster immunization, at which point the mice were just beginning to show clinical signs, they were treated for 2 h with 2.5% sevoflurane dissolved in 100% oxygen, or with 100% oxygen alone. Clinical scores were then monitored for the next 4 weeks. In both groups the incidence of diseases reached 100% at day 23 (Figure 1A). In the control group, clinical scores increased over time (Figure 1B) reaching a maximum value of 2.86 ± 0.46 at day 28; during this time one mouse died at day 25. In the sevoflurane-treated group, clinical scores increased similarly to the control group up until day 25, at which point the scores remained stable until the end of the study (2.14 ± 0. 15). The difference in clinical score development in the sevoflurane-treated mice was statistically different than the control mice (F[9,1] = 1.98, *P* = 0.049, two-way repeated measures ANOVA).

### Sevoflurane reduces leukocyte infiltration

**Figure 2:**
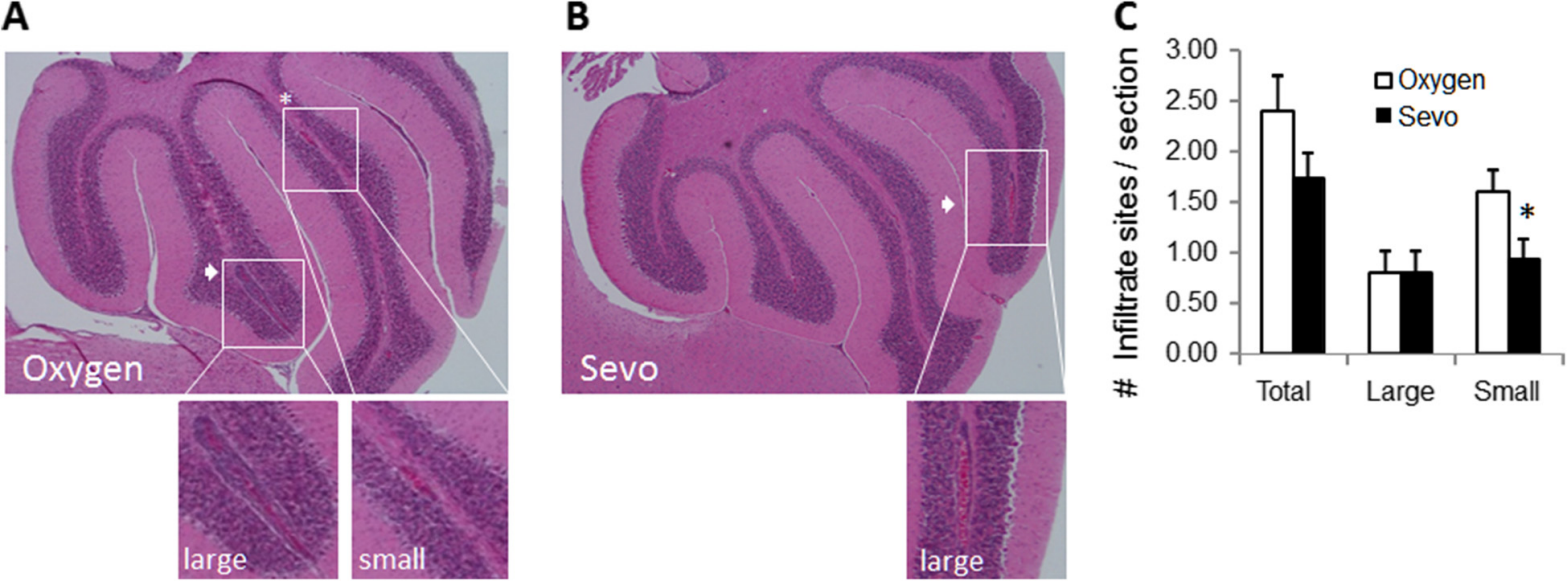
**Sevoflurane reduces leukocyte infiltration in experimental autoimmune encephalomyelitis (EAE).** Representative sagittal sections from (**A**) oxygen-treated and (**B**) sevoflurane-treated mice were stained with hematoxylin and eosin to detect infiltrating cells. Arrows indicate large (>5,000 μm^2^) sites of infiltration; asterisks indicate smaller (<5,000 μm^2^) sites. The lower images show magnified areas as indicated. (**C**) The total number of sites of infiltration was quantified as large or small. Data are mean ± SEM of n = 5 sections per animal, three mice in each of the two treatment groups; *P <0.05 versus oxygen.

At the end of the studies described above, serial sagittal sections were prepared from brains of the oxygen-treated and sevoflurane-treated mice for histological and immunocytochemical analysis. H&E staining to visualize infiltrating cells (Figure 2) showed that while leukocyte infiltration could be detected in the cerebellum of both oxygen-treated and sevoflurane-treated mice, there was a trend to fewer total number of areas of infiltrates in the sevoflurane-treated mice. Classification into larger (>5,000 μm^2^) and smaller (<5,000 μm^2^) areas of infiltrates (Figure 2C) shows that sevoflurane significantly reduced the number of smaller regions containing infiltrating cells.

**Figure 3:**
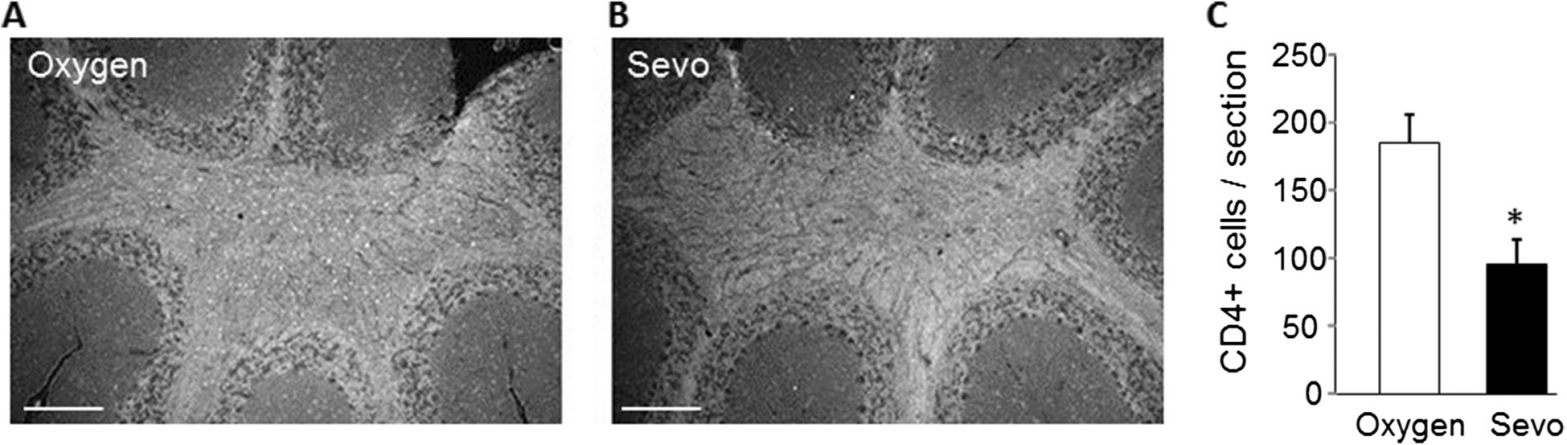
**Sevoflurane reduces CD4+ T cells in the cerebellum of experimental autoimmune encephalomyelitis (EAE) mice.** Representative sagittal sections from (**A**) oxygen-treated and (**B**) sevoflurane-treated mice were stained with antibody against the CD4 receptor to detect infiltrating T cells, which are visible as small cells staining throughout the white matter. (**C**) The total numbers of CD4+ cells per field of view were quantified. Data are mean ± SEM of n = 5 sections per animal, three mice per group; *P <0.05 versus oxygen. Scale bar is 500 μm.

Immunostaining using an antibody to the T cell receptor CD4 (Figure 3) revealed the presence of small CD4^+^ stained cells throughout the brain and large numbers in the white matter of the cerebellum of the oxygen-treated mice. In sevoflurane-treated mice, the number of CD4^+^ stained cells in this area of the cerebellum was reduced by 50% (*P* <0.05).

### Sevoflurane reduces glial activation in EAE

**Figure 4:**
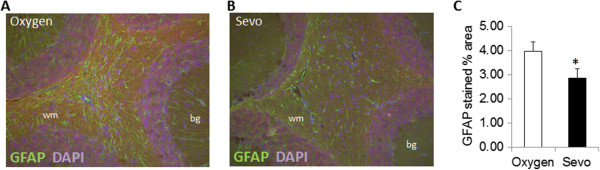
**Sevoflurane reduces glial activation in cerebellum of experimental autoimmune encephalomyelitis (EAE) mice.** Representative sagittal sections from (**A**) oxygen-treated and (**B**) sevoflurane-treated mice were stained with antibody to glial fibrillary acidic protein (GFAP) to detect activated astrocytes. (**C**) The total GFAP+ staining per field of view was quantified. Data are mean ± SEM of n = 6 sections per animal, three mice per group; *P <0.05 versus oxygen. WM, white matter; BG, Bergmann glia.

During EAE, the production of inflammatory mediators from infiltrating T cells leads to the activation of parenchymal glial cells (astrocytes and microglia) throughout the brain and spinal cord. A reduction in T cell numbers could therefore reduce overall glial activation. To test this, we stained serial sections through the cerebellum for the astrocyte specific marker GFAP (Figure 4). In sections from oxygen-treated mice, we observed strong GFAP staining throughout the cerebellum in both the white matter and in the Bergmann radial glial cells (Figure 4A). In the sevoflurane samples (Figure 4B), GFAP staining in the white matter was much less, and only minimal staining of Bergmann glial was observed. Quantitative image analysis confirmed that total GFAP staining in the cerebellum was significantly reduced by 30% in the sevoflurane-treated mice compared to controls (Figure 4C).

### Sevoflurane reduces T cell activation *in vitro*

**Figure 5:**
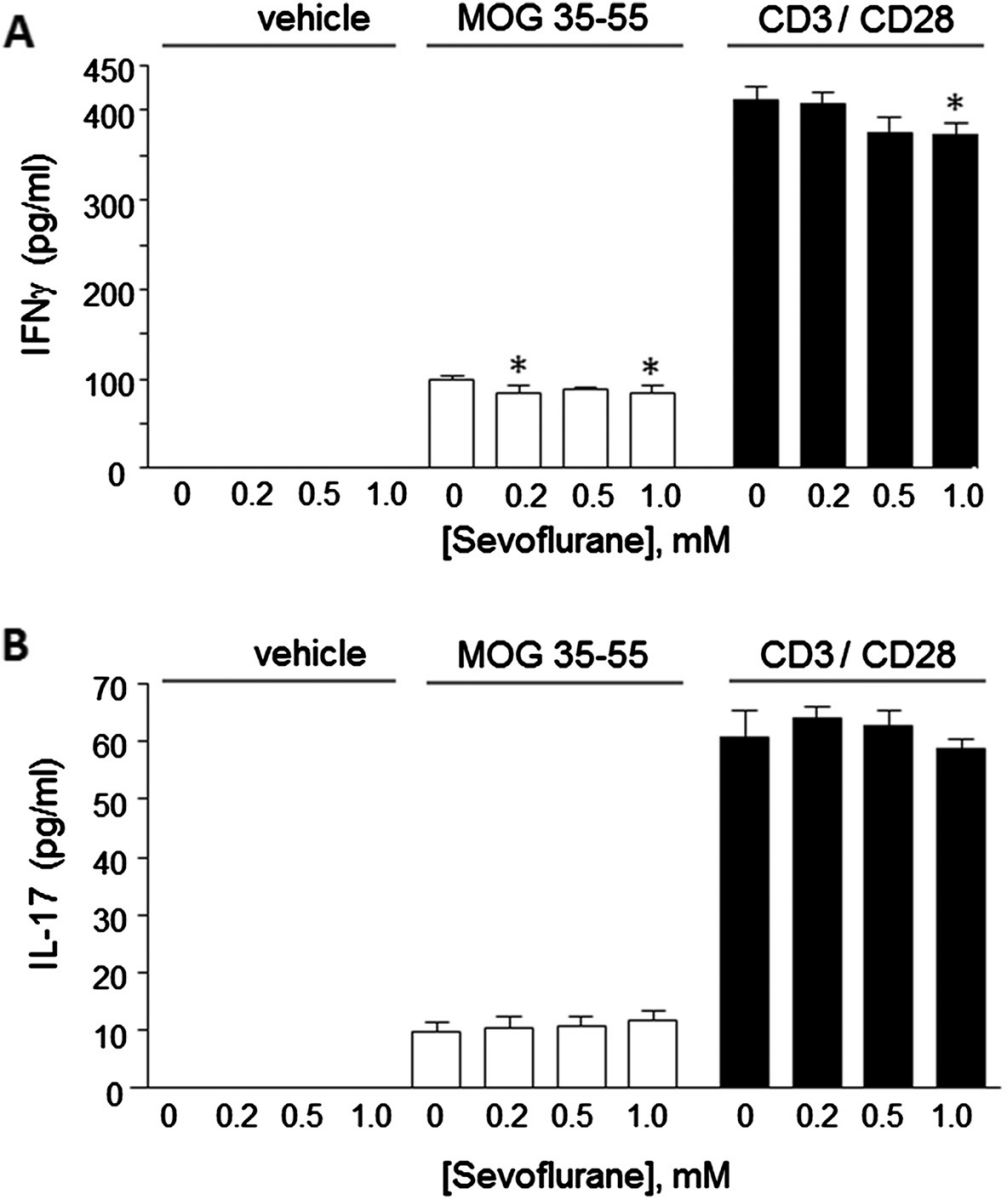
**Sevoflurane modifies T cell cytokine production.** Splenic T cells were prepared from C57Bl/6 mice 10 days after the booster myelin oligodendrocyte glycoprotein (MOG) immunization, and restimulated *ex vivo* with vehicle, with MOG_35-55_ peptide (20 μg/ml), or activated with antibodies to CD3 and CD28 in the presence of the indicated doses of sevoflurane. After 24 h, media levels of (**A**) interferon (IFN)γ and (**B**) interleukin (IL)-17 were determined by specific ELISA. Data are mean ± SEM of n = 4 samples per group; *P <0.05 versus 0 sevoflurane (one-way analysis of variance (ANOVA), Tukey *post hoc* test).

Reduced leukocyte infiltration into the CNS could be due, in part, to suppression of T cell activation by sevoflurane. To test this possibility, splenic T cells were isolated from MOG-immunized mice and activated *in vitro* with MOG peptide or with antibodies to the TCR CD3 and costimulatory receptor CD28 (Figure 5). After 24 h, the T cells produced significant amounts of proinflammatory cytokines IFNγ (Figure 5A) and IL-17 (Figure 5B) compared to non-activated cells, and those levels were greater in the TCR activated cells. In MOG-treated cells, IFNγ levels were reduced by low does of sevoflurane (0.20 to 1.0 mM); in CD3/28-treated cells only 1.0 mM sevoflurane showed a significant reduction. Sevoflurane at these doses did not significantly reduce IL-17 levels in the media.

**Figure 6:**
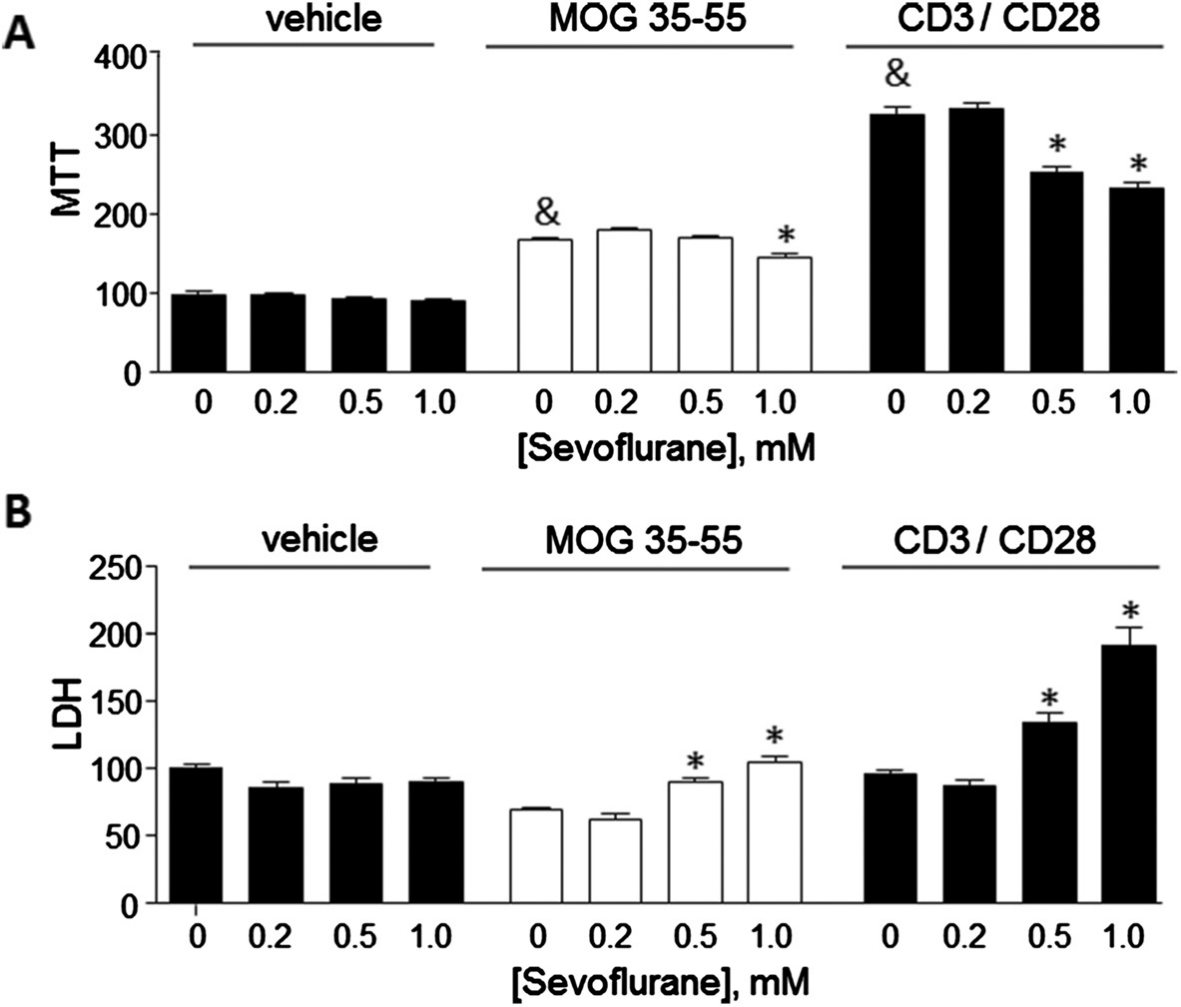
**Sevoflurane reduces T cell proliferation and induces T cell death.** Splenic T cells as described in Figure 5 were assayed after 24 h for (**A**) cell proliferation using the 3-(4,5-dimethylthiazol-2-yl)-2,5-diphenyltetrazolium bromide (MTT) assay; and (**B**) cell death by measurement of lactate dehydrogenase (LDH) in the culture media. The data are mean ± SEM of n = 4 samples per group, and are relative to values measured for vehicle-treated cells with 0 sevoflurane. ^&^*P* <0.05 versus vehicle, 0 sevoflurane (t test); *P <0.05 versus 0 sevoflurane (one way analysis of variance (ANOVA), Tukey *post hoc* test).

Assessment of mitochondrial content using the MTT assay showed a significant increase in cell proliferation due to MOG or to CD3/28 as compared to non-treated cells (Figure 6A). In MOG-treated cells, proliferation was reduced by 1.0 mM sevoflurane, whereas in the CD3/28-treated cells both 0.5 and 1.0 mM sevoflurane reduced proliferation. Measurements of LDH release showed that sevoflurane at 0.5 and 1.0 mM significantly increased cell death in both MOG-treated and CD3/28-treated cells, but not in vehicle-treated cells.

## Discussion

To the best of our knowledge this is the first study to demonstrate neuroprotective effects due to sevoflurane inhalation that attenuated the development of clinical symptoms in experimental autoimmune encephalitis, a well established model of multiple sclerosis. A single exposure of mice to 2.5% sevoflurane for 2 h arrested the continuing development of neurological symptoms related to EAE. This functional neurological protection was associated with a reduction of inflammatory cells within the brain and reduced levels of glial cell activation. In parallel, *in vitro* studies showed inhibitory actions of low doses (between 0.20 and 1.0 mM) sevoflurane on T cell activation, as indicated by reduced accumulation of IFNγ in the culture media, reduced cell proliferation, and increased cell death. These results suggest that low doses of sevoflurane may provide benefit in EAE by suppression of T cell responses.

To place these studies and methodology in a clinical context, patients typically receive sevoflurane, which has a minimum alveolar concentration (MAC) = 1.89, in the range of 1.5% to 2.5%. Measurements of blood levels from patients receiving sevoflurane at 1.5% demonstrated concentrations ranging from 43 to 58 μg/ml or approximately 0.25 mM [29]. Therefore, in our whole animal studies and the *in vitro* studies, concentrations of sevoflurane of 2.5% and 0.5 mM, respectively, are within the clinically relevant range during general anesthesia.

EAE induced by MOG peptide typically results in onset of clinical symptoms beginning at about day 10 after the booster immunization, with a progressive worsening that plateaus near day 25. In this study, the control, oxygen-treated EAE mice reached a maximal clinical score of 2.86 ± 0.46 on day 28 after the booster MOG peptide immunization, which reflects modest to severe neurological injury. In contrast, clinical scores in the sevoflurane-treated mice plateaued at 2.29 ± 0.15 on day 23 after immunization, after which there was no further worsening. These findings suggest that a single exposure to sevoflurane at an early timepoint during the development of EAE can attenuate the ultimate magnitude of neurological damage, although it is not sufficient to reverse the initial damage that has already occurred. Whether longer exposure times, or multiple short exposures to sevoflurane can induce clinical recovery is currently under investigation.

Histological evaluation revealed significant reductions in lymphocytic infiltrates within the cerebellum in the sevoflurane-treated mice. When characterized as to either large (>5,000 μm^2^) or small (<5,000 μm^2^) areas of infiltration, the sevoflurane-treated animals showed a significant reduction in the number of smaller infiltrates. The pathophysiological significance of infiltrate size is not entirely clear but may be due to gradual enlargement of the earlier forming lesion sites. This suggests that sevoflurane is unable to prevent the enlargement of pre-existing sites of infiltrates; but is able to attenuate development of new, smaller lesions.

Our *in vitro* studies point to suppressive actions of sevoflurane on T cells isolated from MOG peptide-immunized mice. This is consistent with previous studies which have described induction of apoptosis, or cell damaging effects of sevoflurane on T cells or lymphocytes, at similar or higher doses, or after longer timepoints. For example, in CD3+ T cells, exposure to 8% sevoflurane, which resulted in a cell culture media concentration of 1.17 mM, induced significant cell apoptosis [10,21]. However exposure to lower doses (2.5%, or approximately 0.40 mM) did not induce apoptosis. In normal peripheral lymphocytes [11] after incubation with sevoflurane at concentrations of 0.5, 1.0, and 1.5 mM it was found that the lowest dose did not increase markers of apoptosis. Cell damaging effects at higher doses of sevoflurane have been reported in other lymphocytes, for example in human B cells, 10 mM sevoflurane (added as 20 μl of the stock 7.5 M sevoflurane into 15.5 ml of saline) induced significant alterations in heme biosynthesis [7]. Our results show that a very low dose (0.20 mM) of sevoflurane could significantly reduce the production of the T helper 1 (Th1) cytokine IFNγ, but that up to 1.0 mM sevoflurane did not reduce IL-17. This suggests that sevoflurane differentially affects distinct T cell subtypes since these two cytokines are produced by Th1 and Th17 T cells, respectively. Further studies using enriched cell populations will be needed to address this possibility.

The ability of sevoflurane to induce T cell apoptosis or modify T cell functionality has been reported several times. As soon as 1 or 2 h after administration of sevoflurane (between 1% to 1.5% in oxygen:air mixture) there was an increase in DNA damage in blood lymphocytes [9]; *in vitro* exposure of normal human PBMCs to sevoflurane (0.5, 1.0, or 1.5 mm) induced apoptosis as soon as 6 h after exposure [11]. Similarly, exposure of healthy adult mice to 3% sevoflurane (1.2 MAC) for 40 minutes reduced the number of circulating PBMCs and splenic B cells, but increased CD4+ lymphocytes in the spleen when measured after 3 days [12], which persisted up to 9 days after anesthesia exposure [16]. The mechanisms by which sevoflurane induces lymphocyte damage are not clear. However, *in vitro* studies using human CD3 T cells showed that sevoflurane suppressed activation of transcription factor AP-1, which plays a role in T cell inflammatory activation [18] as well as inducing apoptosis [10]. These effects also involve protein kinase cascades including activation of the p38 kinase [21]. Interestingly, many of these effects were not observed following exposure to desflurane. In addition to inducing apoptosis, sevoflurane, as well as other inhalation anesthetics, can influence interactions of lymphocytes with other cell types. For example, sevoflurane increased binding of platelets to lymphocytes, and increased expression of P-selectin on the platelets [14]. Sevoflurane inhibited binding of integrin lymphocyte function associated antigen 1 (LFA-1) to its ligand (intercellular adhesion molecule 1 (ICAM-1)) thereby accounting for its ability to reduce inflammatory activation through the ICAM-1 signaling pathway [15]. Whether some or all of these actions of sevoflurane contribute to the beneficial effects observed in the current study are not known; however reductions in numbers, inflammatory state, or migration of T cells (and B cells) would all be expected to be of benefit to diseases such as MS.

Collectively, the results presented in this study suggest that sevoflurane provides protection against T cell mediated neurological inflammation and the subsequent neurological sequelae resulting from T cell infiltration into the CNS and associated activation of parenchymal glial cells. Our immunochemical staining of the cerebellum was limited to CD4+ cells, we cannot rule out that the beneficial effects of sevoflurane are also due in part to reduced infiltration of other cell types such as macrophages or B cells. Measurements of direct effects of sevoflurane on neuronal integrity and axonal damage was beyond the scope of this investigation, but the positive functional protection raises intriguing possibilities for the use of inhaled anesthetic agents in the treatment of neuroinflammatory conditions. These conditions need not be confined solely to primary CNS diseases but viewed in a more global context of inflammation, such as sepsis, that results in wide spread inflammation including in the brain [30-32].

There has been significant interest in the effects of inhalational anesthetics on neuronal function (for example, anesthesia conditioning and protection) but also concern due to increased neuronal apoptosis following exposure to inhaled anesthetics. Widespread concern regarding the effects of inhalational anesthetics on the developing brain was the impetus for the SMARTTots (Strategies for Mitigating Anesthesia-Related neuroToxicity in Tots) program (formerly called SAFEKIDS), and US Food and Drug Administration (FDA)-driven panels to investigate potential long term sequelae of anesthesia in pediatric patients [33]. The concern regarding long term outcomes following exposure to inhalational agents arose on the heels of significant excitement related to widespread demonstration of protective effects of anesthesia preconditioning and even anesthesia post-conditioning in a variety of models including stroke, myocardial infarction and ischemia reperfusion injury. Unraveling the protective effects from injurious effects has proved to be challenging. In a recent review [34], it is apparent that the effects of inhalational anesthetics on the brain are complex and multifactorial and are dependent upon subject age, comorbidities, duration of exposure, concentration and specific anesthetic agent. Collectively, it appears that higher concentrations and longer durations of exposure may be associated with increased neuronal cell death while lower concentrations may confer neuroprotection.

## Conclusions

The current findings demonstrate that a single exposure to the inhaled anesthetic sevoflurane attenuates the progression of EAE, associated with reductions in glial cell activation and T cell infiltration. This raises the possibility that patients with autoimmune-based neurological diseases such as MS could also benefit from a similar treatment.

## Abbreviations

AD: Alzheimer’s disease; ANOVA: analysis of variance; APP: amyloid precursor protein; ASK1: apoptosis signal-regulating kinase 1; ATF2: activating transcription factor 2; CNS: central nervous system; DAPI: 4′,6-diamidino-2-phenylindole; EAE: experimental autoimmune encephalomyelitis; FITC: fluorescein isothiocyanate; GFAP: glial fibrillary acidic protein; IA: inhaled anesthetic; IACUC: Institutional Animal Care and Use Committee; IL-17: interleukin-17; IFNγ: interferon γ; LDH: lactate dehydrogenase; MAC: mean alveolar concentration; MAPKK: mitogen-activated protein kinase kinase; MOG: myelin oligodendrocyte glycoprotein; MS: multiple sclerosis; MTT: 3-(4,5-dimethylthiazol-2-yl)-2,5-diphenyltetrazolium bromide; PBMC: peripheral blood mononuclear cells; PBS: phosphate-buffered saline; PT: pertussis toxin; RRX: rhodamine red-X; TCR: T cell receptor; Th1: T helper 1; Th17: T helper 17.

## Competing interests

The authors declare that they have no competing interests.

## Authors’ contributions

PEP carried out immunostaining for T cells and ELISAs; ROD helped draft the manuscript and discussed the clinical relevance; SK carried out immunostaining for glial cells; AJS immunized and scored mice; RR carried out sevoflurane exposure; GW supervised use of sevoflurane and helped draft the manuscript; DES help conceive of the original study plan; IR contributed to design of the study; DLF conceived the study, carried out data analysis, and drafted the manuscript. All authors read and approved the final manuscript.
